# Integrated workflow for univariate and multivariate evaluation of batch correction reliability

**DOI:** 10.1007/s11306-026-02453-1

**Published:** 2026-07-23

**Authors:** Elfried Salanon, Blandine Comte, Delphine Centeno, Stéphanie Durand, Estelle Pujos-Guillot, Julien Boccard

**Affiliations:** 1 Université Clermont Auvergne, INRAE, UNH, Plateforme d’Exploration du Métabolisme, MetaboHUB Clermont, F63122 Saint-Genès Champanelle, Clermont-Ferrand, France; 2 School of Pharmaceutical Sciences, University of Geneva, Geneva, Switzerland

**Keywords:** Batch correction, Workflow, Reproducibility

## Abstract

**Introduction:**

Assessing batch correction methods remains a major challenge in metabolomics, as no consensus currently exists for a generic and reliable evaluation strategy. Given the strong influence of batch effects on downstream statistical analyses, establishing a robust framework for their assessment is crucial to ensure result reproducibility and validity.

**Methods:**

This study presents a comprehensive workflow that combines innovative numerical indicators and diagnostic plots to assess multiple dimensions of batch correction performance. It relies on a newly developed indicator, the *Batch Conformity Index* (BCI), a multivariate, covariance-aware metric quantifying within- and between-batch variability. Complementary visualization tools, including single and multiblock factorization methods, hierarchical clustering and convex hull representations, provide interpretable global diagnostics. These are complemented by compound-level analyses employing classical univariate metrics such as the coefficient of variation, and intra/inter-batch dispersion indices. The workflow also integrates chemistry-based validation via isotopic ratio consistency to ensure that corrections preserve true biochemical information, enabling the detection of potential overfitting or overcorrection.

**Results:**

The benefits offered by the proposed strategy were illustrated by comparing two widely used correction methods, *i.e.* LOESS and ComBat, applied to a large-scale serum metabolomics dataset. The results highlighted the complementary strengths and limitations of each method, successfully captured by the proposed workflow, thus providing an objective and interpretable basis for method evaluation.

**Conclusion:**

The developed framework offers a unified strategy for evaluating batch correction reliability across multivariate, univariate, and chemical dimensions, representing a significant step toward standardized and reproducible metabolomics data harmonization.

**Supplementary Information:**

The online version contains supplementary material available at https://doi.org/10.1007/s11306-026-02453-1.

## Introduction

Recent advances in analytical techniques, particularly in mass spectrometry (MS) allowed increasing data quality in metabolomics in terms of sensitivity and robustness, opening the door to its large-scale application (Hajjar et al., 2023). However, in MS-based untargeted metabolomics, even if the multiple sources of random error are generally reduced through good laboratory practices, systematic errors, often caused by impaired analytical systems, may affect the quality of collected data. As untargeted metabolomics does not involve absolute quantification, this can hinder the discovery of biomarkers by increasing the risk of false positives or negatives (Leek et al., 2010). These batch effects are usually evaluated via repeated measurements of samples using internal standards or certified reference materials (Broadhurst et al., 2018).

In this context, several approaches and methods have been developed to limit batch effects (Deng et al., 2019; Guo, 2024; Han et al., 2023; Liu et al., 2020; Yu, 1992).

However, there is currently no clear consensus on how to evaluate and compare results obtained with different batch correction methods. Researchers proposing new approaches or assessing existing ones for routine use rely on different evaluation frameworks (Giordan, 2014; Johnson et al., 2007). The first type of strategy aims to assess the degree of bias in the data, either through visual tools, such as Principal Component Analysis (PCA) or t-distributed stochastic neighbor embedding (t-SNE) (Jolliffe & Cadima, 2016; Maaten & Hinton, 2008), or quantitative indicators, including the D-ratio and the coefficient of variation (CV) (Broadhurst et al., 2018; Everitt, 1998). A second evaluation strategy is based on the prediction performance of known biomarkers identified in previous studies. In such cases, a panel of established metabolites is used to assess whether their discriminatory power is preserved or improved after applying a given batch correction method (Giordan, 2014).

However, although these methods coexist, no standardized workflow is currently available for the assessment of newly developed correction algorithms, or those used in routine applications. Furthermore, because batch effects arise from both intra- and inter- batch variation as well as study- and platform-specific artifacts (Broadhurst et al., 2018; Everitt, 1998), their evaluation cannot rely on a single indicator. While multidimensional visualization tools such as PCA provide a global view of performance, they should be complemented by univariate assessments that focus on specific metabolites. Metrics such as CV, intra-group dispersion, or D-ratio offer this metabolite-level perspective. It is also important to assess how well the correction addresses intra- and inter- batch variation (Broadhurst et al., 2018; Salanon et al., 2024). Finally, while most current approaches rely solely on statistical indicators, a complete workflow should also include evaluations from chemical standpoints.

The workflow and tools presented in this work offer a strategy to assess multiple dimensions of batch correction performance. They combine visualization techniques with numerical indicators, supporting both multidimensional and metabolite-specific evaluations. Additionally, the approach integrates statistical tools alongside chemistry-based metrics such as isotopic ratio consistency providing a more comprehensive and reliable framework for method overfitting and overcorrection assessment.

## Materials and methods

### Preliminary method development: the Batch Conformity Index

The proposed *Batch Conformity Index* (BCI) is based on the Mahalanobis distance (Li et al., 2019). Providing a multivariate indicator, BCI is relevant for assessing batch effect correction in metabolomics and other fields requiring data harmonization, where variations introduced by external conditions must be minimized, while preserving meaningful biological differences.

- **Definition and mathematical formulation**

The BCI can be computed as a weighted average or median of Mahalanobis distances, capturing the dispersion of observations around a reference point, as follows (1):

1 $$ BCI = \frac{{\mathop \sum \nolimits_{i = 1}^{n} w_{i} .D_{i} }}{{\mathop \sum \nolimits_{i = 1}^{n} w_{i} }} $$

where ${D}_{i}$​ represents the Mahalanobis distance of observation *i* to the barycenter, defined as the median of the reference group on each variable, building the global median QC for example when the estimation is about QCs. The weighting factor ${w}_{i}$ allows for flexible adjustments, incorporating measurement reliability or other relevant features. The Mahalanobis distance itself is computed as (2) (Li et al., 2019):

2 $$ D\left( {x,\mu } \right) = \sqrt {\left( {x - \mu } \right)^{T} \mathop \sum \limits_{{}}^{ - 1} \left( {x - \mu } \right)} $$

where *x*, is an observation, *μ * is the barycenter, and *Σ * is the covariance matrix, which can be adapted to different assumptions about the data structure. Unlike conventional distance metrics, this formulation accounts for the correlation between variables, making it well suited for high-dimensional, structured datasets such as those collected in metabolomics. To provide a robust estimation, the BCI uses the median as reference, ensuring that extreme values do not disproportionately influence the measure. Furthermore, when the covariance matrix is ill-conditioned or singular, shrinkage techniques are applied to ensure reliable inversion. Finally, in the present work, the BCI is computed using equal weights, corresponding to a simple average of Mahalanobis distances. This choice ensures interpretability and avoids introducing additional assumptions. However, alternative-weighting schemes can be considered, such as weights proportional to the relative frequency of each batch, in order to account for imbalance between batches. Regarding covariance estimation, the empirical covariance matrix is used by default. To ensure numerical stability and avoid singularity issues, a shrinkage estimator is applied when necessary. When enhanced robustness to outliers is required, more advanced estimators such as the Minimum Covariance Determinant (MCD) can be employed. These approaches offer increased tolerance to extreme observations by relying on robust estimates of location and scatter. However, given the controlled nature of the QC data used in this study and to preserve computational efficiency and interpretability, such alternatives were not implemented in the present workflow.

- **Properties and justification**

A key BCI property is its invariance to linear transformations, inherited from the Mahalanobis distance. This ensures that comparisons between datasets remain meaningful even when scaling or affine transformations are applied. Additionally, BCI is sensitive to covariance variations, allowing it to capture dependencies between variables that might otherwise be overlooked with simpler distance metrics. The measure is also stable in the presence of extreme values, particularly in its robust version, which uses the median as a reference and applies shrinkage-based or robust estimations of the covariance matrix to mitigate the influence of outliers.

### Workflow for batch correction assessment

**Fig. 1:**
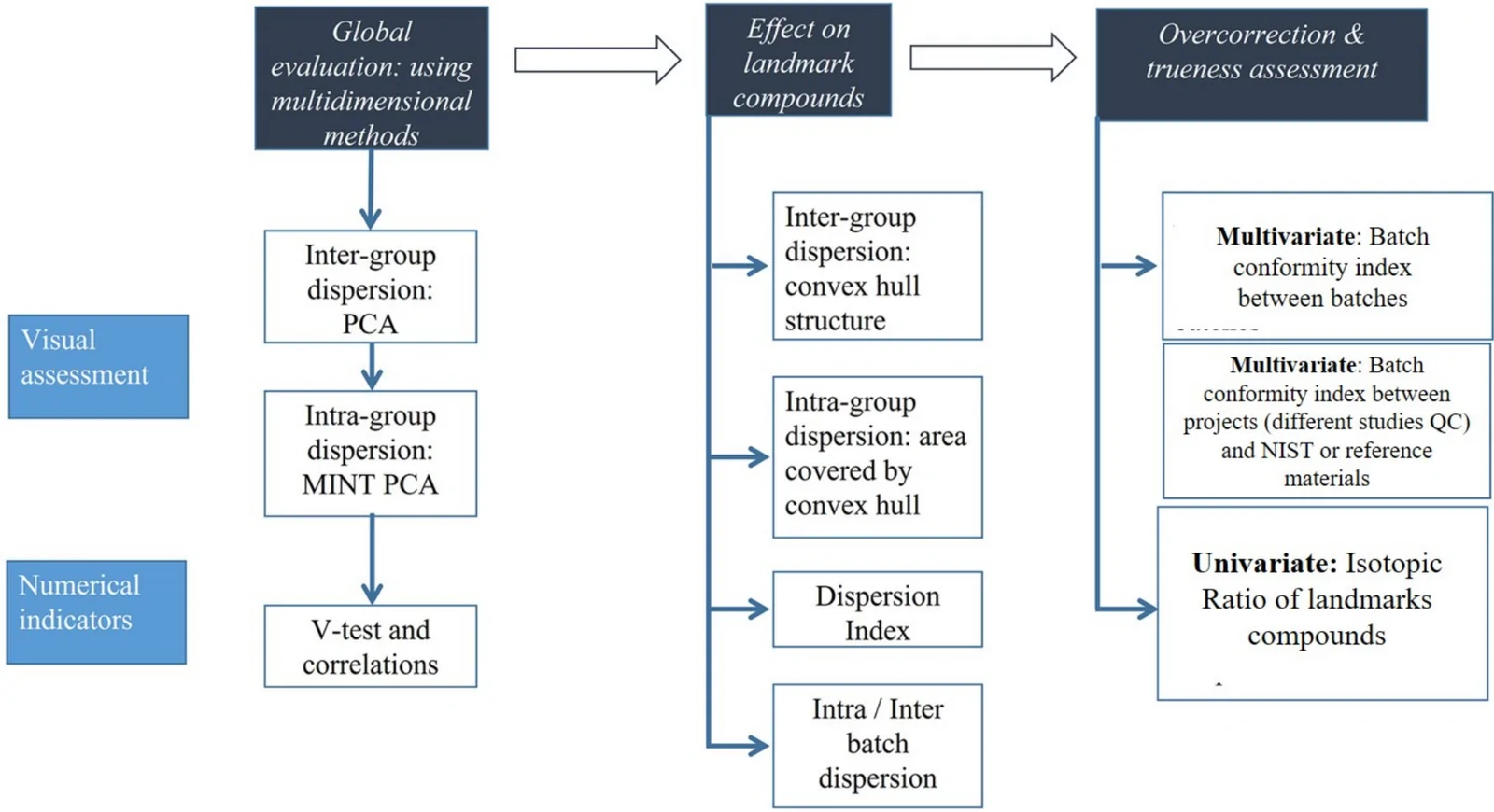
Comprehensive workflow for assessing the reliability of batch correction methods

The proposed evaluation, of batch effect as presented in Fig. 1 is a multistep approach, combining multidimensional and univariate tools.

*Step 1: Global evaluation: using multidimensional methods (Visualization and numerical assessment) before and after correction*

Principal Component Analysis (PCA) (Jolliffe & Cadima, 2016) and Multivariate INTegrative (MINT) PCA (Rohart et al., 2016, 2017) are used for multidimensional assessment to visualize inter- and intra- batch effects. Unit variance scaling was applied in both cases. Multivariate INTegrative Principal Component Analysis (MINT-PCA), introduced by Kim-Anh Lê Cao and colleagues (Rohart et al., 2016), is an extension of classical PCA designed for the integrative analysis of data originating from multiple studies or batches. Unlike standard PCA, which maximizes overall variance without accounting for the source of the data, MINT-PCA explicitly incorporates group structure (e.g., batches or cohorts) into the model. Its objective is to extract components that capture variation shared across groups, thereby emphasizing reproducible biological signals rather than technical variability. In the context of metabolomics, this property is particularly relevant, as batch effects can dominate the principal components in classical PCA, leading to representations primarily driven by analytical artifacts. By contrast, MINT-PCA constrains the decomposition to highlight patterns that are consistent across batches, improving the interpretability of multivariate structures and enabling a more reliable assessment of batch correction performance. In practice, PCA remains a purely unsupervised exploratory tool suited for initial data visualization, whereas MINT-PCA provides a more robust, integrative framework for evaluating inter-batch consistency and the preservation of meaningful biological variation after correction.

PCA was complemented with hierarchical clustering to assess clustering consistency before and after correction. Euclidean distance was used as the dissimilarity metric, and hierarchical clustering was performed using Ward’s linkage method (Ward.D2), which minimizes within-cluster variance. The number of clusters was determined based on dendrogram inspection and the analysis of within-cluster inertia (elbow criterion). The expected outcome is that the observed clustering should not be driven by batch effects. Henceforth, after effective correction, cluster assignments should be independent of batch labels. This was quantitatively evaluated using a chi-square test of independence between batch labels and cluster assignments. A strong association before correction, followed by a reduced or non-significant association after correction, indicates effective batch effect mitigation. The strength of the association was further quantified using Cramér’s V, a standardized measure of association between two nominal variables derived from the chi-square statistic. Those indicators were complemented with the V-test to compare the proportion of a batch within a cluster to its overall proportion in the dataset, scaled by the standard error. Under the null hypothesis, the V-test follows approximately a standard normal distribution. A threshold of |V|> 1.96 was used to determine statistical significance at the 5% level.

In addition, using the barycenter of each batch as reference, the BCI of the different measurement points provides a multidimensional numeric evaluation of the intra-batch effect, while considering the median of batch values provides a global assessment. BCI is also used for inter-batch evaluation using the barycenter of the different batches and considering the first batch as reference. As proposed recently, Mahalanobis distances of samples to their batch barycenter can be efficiently visualized using convex hulls (Salanon et al., 2024). It provides a simple graphical evaluation of the global dispersion within batch before and after correction.

*Step 2: Targeted assessment of landmark compounds*

Prior choice of the landmark compounds must be done based on expert knowledge to cover the range of physicochemical diversity of the detected metabolites. To ensure the reliability of the results obtained, these compounds have to be annotated features, as level 1 or 2 (Sumner et al., 2007), as well as being detected within the linear range of the instrument. This allows their chemical formulae and isotopic patterns to be measured without bias. Based on these landmark compounds, univariate assessment aims at evaluating the effect of the correction in specific chromatographic areas. The coefficient of variation and the inter-group dispersion (Kloet et al., 2009; Salanon et al., 2024) are used for numeric assessment of the inter-batch correction, while the inter-group dispersion and the dispersion index provide a numeric estimation of the inter-batch effect (Salanon et al., 2024).

Intra-group dispersion (IntraD): indicator of variability within a batch, based on the median of the areas of the convex hulls of the Quality Control (QC) samples, reflecting the dispersion of the measured intensities independently of any parametric hypothesis.

Inter-group dispersion (InterD): indicator of variability between batches, defined as the dispersion gradient between groups, combining differences in injection orders and intensities to quantify analytical drifts.

Dispersion index (D-Index): synthetic indicator of the ratio between intra- and inter- batch variability, making possible to characterize the overall structure of analytical variability using a single value.

A visual evaluation can also be performed using convex hulls for measuring bivariate dispersion. It allows displaying the correction effect on the inter- and intra- batch variability via the landmark compounds in a reliable way.

*Step 3: Overcorrection & agreement with theoretical isotopic values assessment*

In the context of evaluating batch correction methods, it is essential not only to quantify how well they reduce batch-related variability, but also to verify that they do not overcorrect the data. One way to monitor this can be done by using BCI, calculated as the distance between QC samples and a reference material (e.g., NIST SRM 1950 for plasma). The expected behavior after correction is a decrease in BCI between QCs across batches, while maintaining (or even increasing) the distance between the average QC signal and the reference material. In short, the correction should group QCs together, but not distort the biological information present in the data. To further evaluate potential overcorrection, we used the natural isotopic ratios of a set of landmark compounds (determined from peak picked data using the M/M + 1 ratio for ^13^C isotopologues), which have known theoretical values. These serve as internal standards to assess how much the correction respects true chemical information. For each compound, we computed the absolute error and the relative error as follows:

$$ Absolute Error = \left| {Measured Ratio - Theoretical Ratio} \right| $$

$$ {\mathrm{Re}} lative Error \left( \% \right) = \frac{{\left| {Measured Ratio - Theoretical Ratio} \right|}}{Theoretical Ratio} \times 100 $$

Using both metrics is key as absolute error captures the difference from the theoretical value in raw units, while relative error flags when an apparently small deviation is actually large in proportion to the expected ratio (which matters especially for low-abundance compounds). As a rule of thumb, we considered that a relative error > 5% indicates a potentially meaningful deviation. These thresholds are consistent with typical tolerances in isotopic quantification in biological matrices for metabolomics, using QToF instruments. Moreover, systematic bias was evaluated using paired Wilcoxon signed-rank tests comparing observed and theoretical isotope ratios. In addition, the variability was quantified using the absolute deviations from theoretical values. Combining this approach with BCI offers an efficient strategy to detect whether correction methods preserve chemical information or, on the contrary, introduce distortions. In that sense, isotopic ratios are more than a validation step as they offer a practical, compound-level tool to detect and avoid overcorrection of batch effect.

### Application of the workflow in the comparison of two batch correction methods

- **Presentation of the use case**

Two batch effect correction methods were evaluated for this use case, namely the LOESS-based (Dunn et al., 2011) and the ComBat correction (Johnson et al., 2007). While the former is one of the most widely used QC-based approaches in metabolomics, the latter provides a data-driven correction without the need of QCs. Based on the QC samples, LOESS involves the use of a local regression to adjust a model of the drift, which is then applied for data correction. It has the advantages of considering potential non-linearity. On the other hand, the ComBat method includes the use of empirical Bayes methods and prior knowledge (when available) to adjust a noise free model using linear regression (Johnson et al., 2007). It is important to note that the minimum number of QCs requested for batch correction can vary from one method to another. From a more technical perspective, some of the indicators used in this work are based on convex hulls that cannot be computed with less than 3 QCs per batch.

- **Dataset used for the application**

The goals of this use case were to assess the ability of the workflow to capture different dimensions of the batch effect and evaluate its correction in a context of large-scale metabolomics. The use case was conducted using pool QC samples from a predictive metabolomics study of neurological and metabolic toxicities induced by breast cancer treatment (Piffoux et al., 2024). Briefly, this dataset was obtained from 108 pool QC samples made of the mix of 992 serum samples, and analyzed through 9 randomized batches using ultra-high performance liquid chromatography coupled with high-resolution Quadrupole Time-of-Flight (QToF) mass spectrometry (UHPLC-HRMS). Full details of sample preparation and analysis are published by Piffoux et al. (Piffoux et al., 2024). A total of 1,826 features were extracted and included in the current study. The data processing details, were published by Piffoux et al. (Dunn et al., 2011). Data were processed under the Galaxy web-based platform Workflow4Metabolomics (W4M) (Giacomoni et al., 2015). First, raw data were extracted using XCMS (Smith et al., 2006; Tautenhahn et al., 2008). Quality checks and signal drift correction were then carried out using the algorithm described by van der Kloet et al.(Kloet et al., 2009) based on the use of pooled QC samples and the LOESS method with a span of 1 for the whole dataset, to yield a data matrix containing retention times, masses, and peak intensities that have been corrected for batch effects. Combat was implemented using the sva packages (Leek et al., 2012) in R without external covariate.

## Results & Discussion

Application of the workflow in the comparison of two batch correction methods

### Multivariate assessment

#### Inter-batch assessment

- **Visual evaluation with PCA**

**Fig. 2:**
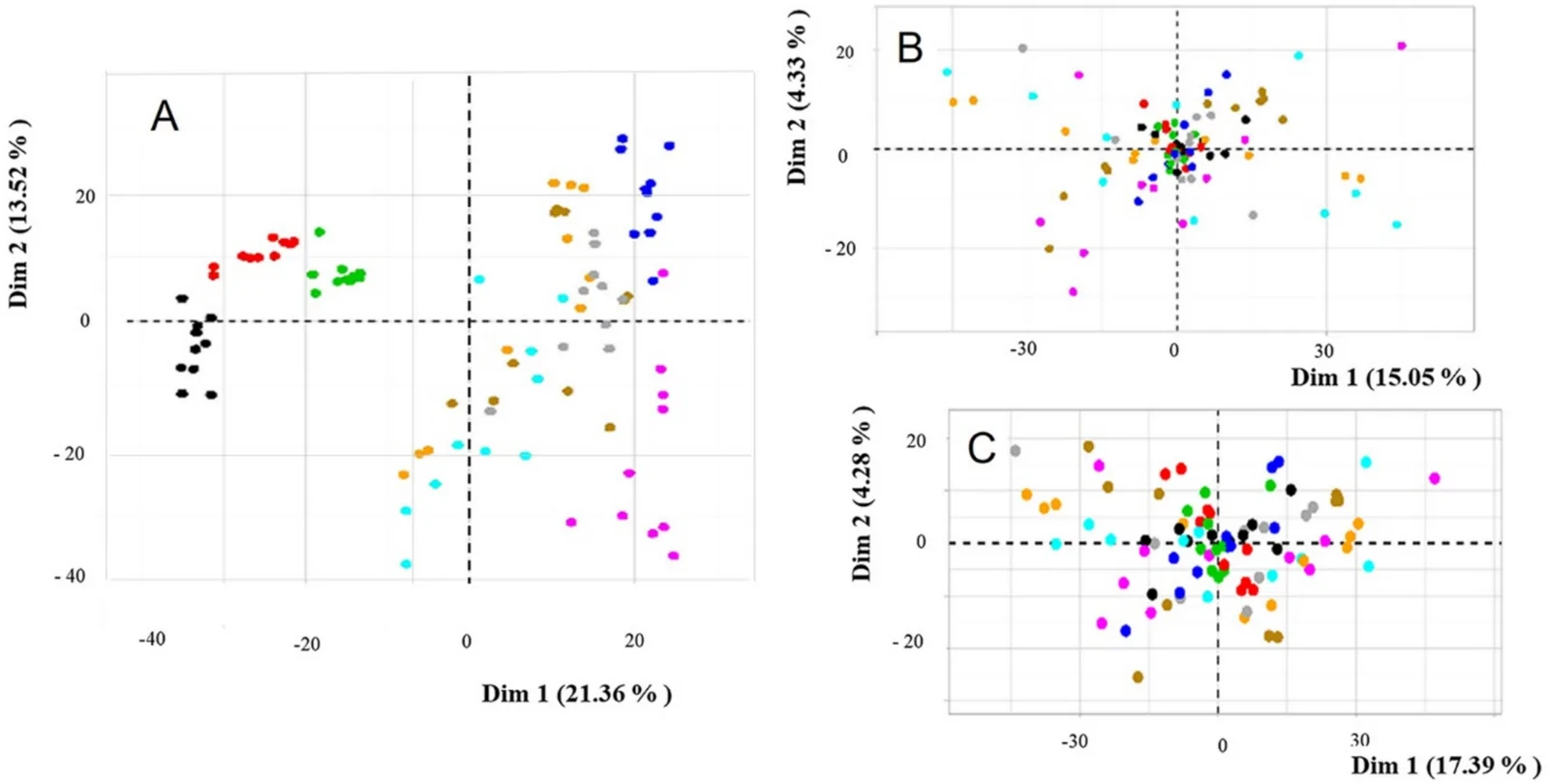
PCA score plot using the QC samples analyzed across nine batches on a large-scale study. *a: Without batch correction, b: After application of the LOESS batch correction, c: After application of the ComBat correction on the data. The different colors represent the different analytical batches*

QCs linked with different batches appeared to be clearly separated on the PCA score plot (PC1 *vs.* PC2) before batch effect correction (Fig. 2a). Batch QCs were well centered after the LOESS correction (Fig. 2b) and the same trends were observed with the ComBat-based correction (Fig. 2c). However, a less uniform distribution was obtained with the LOESS method. Moreover, the correlation of the Batch and the injection order *(r* = *0.61 before correction vs. -0.02 after LOESS & -0.01 after ComBat)*, which were important on the first two principal components, could not be observed anymore after correction with both methods. This was followed by a reduction of the proportion of variance explained by those two dimensions *(Dimension 1* = *21.36% before correction vs. 15.05% after LOESS & 17.39% after ComBat; Dimension 2* = *13.52% before correction vs. 4.33% after LOESS & 4.28% after ComBat)*. This showed a global improvement in the data structure by reducing the inter-batch distance. However, knowing that PCA offers a relative evaluation of the variability contained in the data, it should be considered as multidimensional assessment and a first exploration that needs to be completed with other criteria.

- **Hierarchical clustering**

**Fig. 3:**
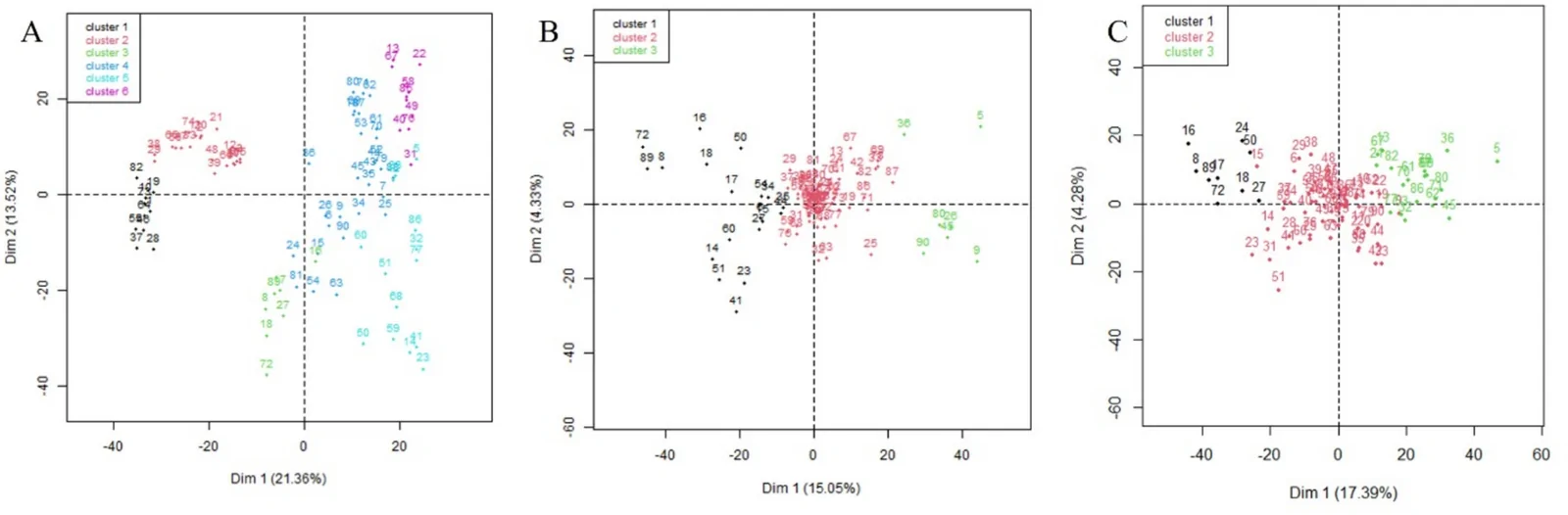
Projection in two dimensions of the results of the Hierarchical Cluster Analysis, using QC samples analyzed within the nine batches. ***a****: Without batch correction, ****b****: After LOESS correction, ****c****: After ComBat correction. The different colors represent the different batches*

Figure 3 presents the clustering using hierarchical cluster analysis after PCA using 5 components to focus on major trends in the data without introducing noise. As presented in Fig. 3a, before correction, cluster 1 was associated with batch 1 *(Value-test (V-test)* = *7.37),* while batches 2 & 3 were linked to cluster 2 *(V-test* = *5.53).* Cluster 3 was composed of batches 9 & 8 with a v-test of 2.17 and the clusters 5 & 6 included respectively batch 5 *(V-test* = *6.38)* & batch 4 *(V-test* = *7.37).* Three clusters were identified after LOESS correction (Fig. 3b). The first cluster was linked to batch 8 *(V-test* = *2.09).* Batch 9 was associated with cluster 3 (*V-test: 3.02*). The other batches were correlated to cluster 2. The ComBat-based batch correction revealed three clusters, with batch 5 being the only associated to cluster 3; all the others were found to be a mix of all the batches. Moreover, the association between batch and cluster assignments was assessed using chi-square tests and quantified using Cramér’s V. A very strong association was observed before correction (p < 2.2e-16, V = 0.88). It markedly decreased after LOESS correction (p = 3.15e-05, V = 0.52) and was no longer statistically significant after ComBat correction (p = 0.065), although a moderate effect size remained (V = 0.37), indicating a substantial but incomplete reduction of batch effects. This analysis revealed a reduction of the batch effect, with an improvement of the homogeneity of the clusters. However, some trends could still be observed for batches 8 and 9 after applying the LOESS methods, and batch 5 after the ComBat method. This could raise some questions about the specificities of those batches in order to identify any potential event that could have occurred during the data generation process. While classical PCA was limited to a visual assessment of the inter-batch changes before and after correction, hierarchical clustering allowed groupings to be more objectively evaluated with respect to batch effect or to a potential biological trend. This could be investigated via the membership of the different batches to the clusters, highlighting others sources of effect, which may be of interest.

- **Numerical evaluation**

Table 1 presents BCI values before and after batch correction calculated on QC samples. The inter-batch correction corresponds to the multivariate assessment of the distance of the barycenter (median) of each batch to the first. Before correction, the median distance was 480.47, while BCI values of 16.53 and 16.63 were observed after correction by LOESS and ComBat methods, respectively. This measurement gives an objective evaluation of the multivariate distance (involving > 1,800 features) of the different batches to the first. This value is meant for comparison relatively to the value before correction. In this use case, there was an improvement of the global inter-batch comparability shown by the decrease of the distance between the different batches to the reference. This is an expected behavior of the correction methods aiming to reduce the difference between the barycenter of the different batches. Moreover, the comparison of the two batch correction methods revealed that they globally performed equally. This is consistent with the results of the visual assessment of the inter-batch variation, showing that both correction methods reduced significantly the inter-batch effect.

**Table 1:** Inter- and intra- batch BCI, before and after correction, using the 108 QC samples analyzed within nine batches on a large-scale study

	Intra-Batch BCI				Inter-Batch BCI	
Batch	Before correction	LOESS	ComBat	Before correction	LOESS	ComBat
Batch1	8.25	8.23	8.27			
Batch2	8.22	7.96	8.10	436.23	15.56	13.98
Batch3	8.10	7.49	8.12	521.44	15.92	15.97
Batch4	8.21	7.24	8.21	477.64	16.62	16.21
Batch5	8.25	7.76	8.37	456.66	23.59	17.47
Batch6	8.39	7.99	8.37	526.62	16.44	16.91
Batch7	8.68	6.85	8.67	531.08	15.98	16.35
Batch8	8.22	7.06	8.24	414.25	19.96	21.20
Batch9	7.06	7.19	8.64	483.30	20.18	18.71
Global Intra-batch BCI	8.22	7.49	8.27			
Global Inter-batch BCI				480.48	16.53	16.63

#### Intra-batch assessment

**Fig. 4:**
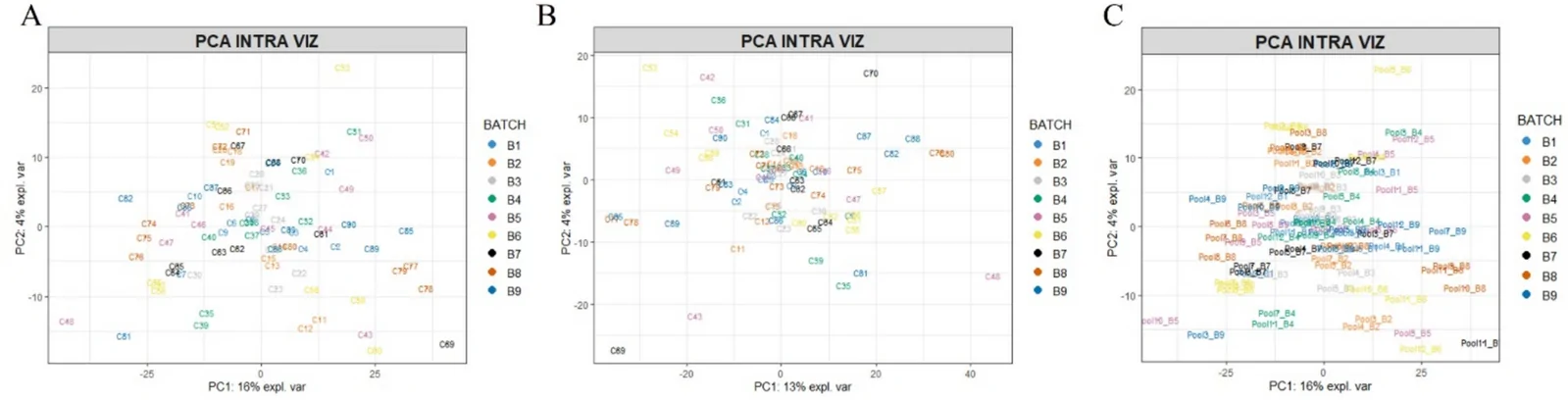
MINT PCA score plots using QC samples analyzed within the nine batches on a large-scale study. ***a****: Without batch correction, ****b****: After LOESS batch correction and ****c****: After ComBat correction. The different colors represent the different batches*

Figure 4 presents the multivariate evaluation of the intra-batch dispersion using MINT PCA. Only little improvement was observed after correction, but LOESS seemed to generate more spread observations scores (Fig. 4b). Numerical estimators were further investigated to better evaluate the dispersion and complement this first visual assessment.

As presented in Table 1, the multivariate assessment of the intra-batch dispersion using the median BCI to their barycenter implying all the variables (> 1,800 features), revealed little differences between the batches except for batch 9, which had a particularly low value of intra-batch BCI. LOESS correction showed a lower intra-batch BCI, while ComBat did not seem to provide any major improvement. The expected behavior for the intra-batch assessment is a reduction of BCI in both the different batches and the global intra-batch value. This decrease revealed a multivariate improvement of the distance of each QC sample to the barycenter of its batch, showing an enhancement in batches harmonization.

**Fig. 5:**
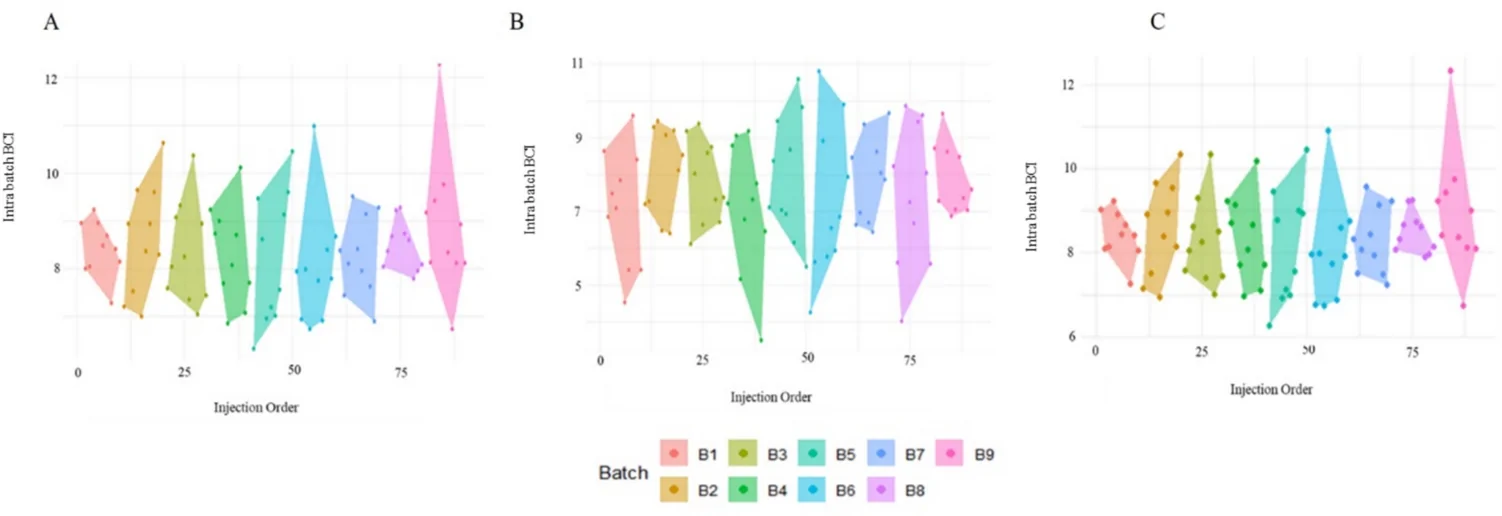
Intra-batch BCI convex hulls, before and after correction, using QC samples analyzed within the nine batches. ***a****: Without batch correction, ****b****: After LOESS correction and ****c****: After ComBat correction. The different colors represent different batches*

Moreover, analyzing the shapes of convex hulls of the different batches (Fig. 5) revealed that ComBat largely preserved the dispersion structure before and after correction, whereas LOESS correction tended to modify it. This conservative behavior of ComBat can be advantageous in preserving the underlying biological structure of the data, as it relies on adjustments of mean and variance (Johnson et al., 2007). However, in the specific context of batch correction based on repeated QC samples, such preservation may indicate a limited ability to correct batch-specific distortions, since effective QC-based correction is expected to reduce intra-batch variability and harmonize batch structures. In contrast, LOESS, which explicitly models QC trends through local regression, may better capture and correct such batch-specific variations, at the cost of altering the dispersion structure.

This assessment is multivariate and is intended to give a global idea of the improvement of the quality. However, targeted evaluation using individual metabolites, *i.e.* landmark compounds, constitutes an interesting complement to evaluate some specific aspects in a univariate way.

### Univariate assessment using landmark compounds

#### Inter-batch effect

Table 2 presents the coefficients of variation as well as the inter-group dispersion of the selected landmark compounds. Results showed a significant decrease in those indicators after correction using both correction methods. Based on the coefficient of variation, LOESS outperformed ComBat except for one amino-acid (L-threonine). This is consistent with the results of the inter-group dispersion which revealed the same trends for most indicators. These results are also consistent with the BCI numeric assessment in a multivariate context, showing that LOESS outperformed ComBat for inter-batch correction. This is further confirmed by the visual assessment of landmark compounds bivariate dispersions. Indeed, while ComBat and LOESS aim to correct for inter-batch differences, ComBat tends to increase batch-wise dispersion in order to get an alignment of the mean intensity of the different batches, while the intra-batch dispersion was still very large in the different batches.

**Table 2:** Univariate assessment of the landmark compounds using the coefficient of variation, the intra-group dispersion, inter-group dispersion and the dispersion index before, and after correction (with Combat and LOESS)

Metabolites	Intra-group dispersion			Dispersion Index			Inter-batch dispersion			Coefficient of variation of measured intensities (%)		
	Before correction	LOESS	ComBat	Before correction	LOESS	ComBat	Before correction	LOESS	ComBat	Before correction	LOESS	ComBat
Isovaleryl-L-carnitine	1448.0	932.9	1267.6	2.2	24.4	17.3	672.4	37.2	72.3	16	9.9	10.8
L-Tyrosine	111,783.2	105,240.4	119,668.1	4.4	5.3	11.5	25,433.5	19,904.5	10,364.8	19	11.4	11.4
Hexanoyl L-carnitine	1956.0	1763.4	2144.0	2.1	26.9	19.4	950.1	64.4	109.7	21	14.8	15
L-Histidine	2889.6	2246.1	2655.1	3.3	5.8	17.0	865.3	386.2	155.2	10.3	6	6.4
Theophylline	6526.0	4750.7	7658.8	1.5	5.8	25.4	4496.3	815	744.7	11.4	5.5	6.6
L-Valine	969,595.6	1,038,837.6	1,046,562.6	4.5	32.3	15.4	214,459.9	32,116.3	68,083.5	12.5	7.8	8.5
L-Tryptophan	55,238.0	50,252.1	51,544.6	1.9	15.3	3.9	28,782.6	3290.9	13,383.6	13.8	8.3	9.6
Caffeine	45,772.0	25,869.4	37,157.7	2.0	3.8	6.0	22,826.1	6744.8	6233.1	16.7	9.9	9.6
Uric acid	51,623.6	54,222.6	72,398.5	1.0	1.2	10.3	50,902.4	45,419.5	12,975.6	18.2	17	12.1
L-Leucine	57,322.4	48,727.2	58,905.8	1.5	7.7	1.3	37,435.1	6328.5	44,886.9	19.6	11.1	14.8
Creatinine	88,327.6	39,434.3	86,291.9	8.1	11.9	16.8	10,916.4	3305.7	5138.3	20.4	8.8	9.4
L-Glutamic acid	6540.8	4539.1	6973.1	1.7	8.8	6.8	3865.7	514.9	1030.2	21.2	7.3	9.3
Decanoyl L-carnitine	8693.6	5159.8	8400.3	4.0	22.2	21.0	2194.3	231.2	398.2	21.9	12.8	15.2
L Methionine	72,570.8	67,882.9	4657.5	2.0	1.9	8.9	36,473.2	36,443.8	525	24.1	19.6	23.5
Myristoyl L-carnitine	1400.4	1110.8	1490.7	2.7	39.8	46.5	525.6	26.9	31.1	27.1	15.1	17.8
Phenylacetyl L-glutamine	9936.0	4056.6	2871.0	1.7	26.9	25.4	5926.4	149.8	330.7	27.9	9.7	11.8
L- Lysine	57,539.6	52,685.1	110,698.4	0.8	0.4	2.0	68,703.2	132,214	56,210.7	28.7	15.1	15.7
Creatine	91,456.8	37,553.6	82,437.1	3.2	6.7	15.8	28,386	5606	5211.1	30.7	11.3	14.1
Lyso PC(P-16:0/0:0)	6618.0	6769.8	8337.1	1.2	12.4	8.8	5353.8	546.8	948.8	34.6	19.8	23.5
L Threonine	2815.2	2841.9	91,934.3	4.0	16.3	21.5	709.2	172.9	4268.2	37.9	17.5	6.9

#### Intra-batch correction

Table 2 presents the intra-group dispersion and the dispersion index of the landmark compounds, showing that intra-group dispersion was globally decreased after correction. However, it should be noted that some values increased after correction. Moreover, intra-group dispersion was globally lower after LOESS correction compared to Combat. These findings are consistent with the results of the multivariate assessment regarding intra-group dispersion. The expected behavior is a decrease in the value of the intra-group dispersion showing an improvement. However, the increase in the dispersion index for most of the landmark compounds indicated that both correction methods led to better results in the correction of the inter-group than in the intra-group effect. Indeed, the intra-batch variability is more strongly connected to the structure of the analytical shift resulting from the analytical sequence, while the inter-batch effect is more related to global differences, making it more straightforward to correct.

These findings were confirmed by the visual representations of the bivariate dispersion of landmark compounds in Supplemental Fig. 1. While there was a decrease in the inter-batch distance, the intra-batch dispersion of some batches increased in order to have a global alignment. For example, the intra-batch variability of uric acid was preserved without improvement or deterioration after LOESS correction, while increased intra-batch dispersion was observed after ComBat. The same trends were observed for lysine and L-methionine.

#### Effect of the correction on the isotopic ratios

Table 3 presents the isotopic ratios of several landmark metabolites before and after correction using LOESS and ComBat methods, considering only measurements above the S/N > 10 threshold. The goal was to assess how batch correction algorithms preserve chemically known value, using theoretical natural isotope ratios as reference values.

**Table 3:** Average intensities, isotopic ratios, and their standard deviations, before and after correction, using the QC samples analyzed within nine batches. Filtered by intensities

Landmark compounds	Isotopic Ratios*				Difference** (and percentage***) of the Isotopic Ratios* to the theoretical value			Average intensities			Standard Deviations		
	Theoretical	Without correction	LOESS	ComBat	Without correction	LOESS	ComBat	Without correction	LOESS	ComBat	Without correction	LOESS	ComBat
L-Proline	**6.00**	**6.85**	**6.86**	**7.13**	**0.85(14.2)**	**0.86(14.3)**	**1.13(18.8)**	5,016,879.31	5,057,731.28	977,707.24	0.25	0.18	0.21
L-Tryptophan	12.80	*14.55*	*14.61*	*14.56*	*1.75(13,7)*	*1.81(14.1)*	*1.76(13.8)*	2,467,803.36	2,463,565.55	2,463,281.09	1.53	1.33	0.67
L-Tyrosine	10.30	*11.49*	*11.46*	*11.49*	*1.19(11.6)*	*1.16(11.3)*	*1.19(11.5)*	1,648,241.47	1,648,516.32	1,647,534.54	0.42	0.49	0.40
Creatinine	**5.50**	**5.34**	**5.38**	**6.85**	**-0.16(-2.9)**	**-0.12(-2.2)**	**1.35(24.5)**	1,326,372.13	1,329,412.24	5,025,833.73	1.16	1.16	1.15
L-Methionine	**6.80**	*7.06*	*7.06*	*7.07*	*0.26(3.8)*	*0.26(3.8)*	*0.27(4.0)*	1,198,202.47	1,201,474.60	1,196,698.18	50.61	35.85	3.00
Pipecolic acid	**7.10**	**7.30**	**7.31**	**5.22**	**0.2(2.8)**	**0.21(-2.9)**	**-1.88(-26.5)**	978,320.80	982,817.45	875,383.14	2.79	2.27	1.59
Creatine	**5.60**	**5.46**	**5.50**	**8.13**	**-0.14(-2.5)**	**-0.1(-1.8)**	**2.53(45.2)**	873,001.58	878,834.40	461,399.23	1.06	1.27	1.40
L-Lysine	7.50	*7.23*	*7.19*	*7.07*	*-0.27(-3.6)*	*-0.31(-4.1)*	*-0.43(-5.7)*	724,408.40	722,198.92	1,196,698.18	1.69	1.58	1.87
Caffeine	10.30	*10.85*	*10.86*	*10.85*	*0.55(5.3)*	*0.56(5.4)*	*0.55(5.4)*	594,063.33	599,396.73	593,703.24	0.78	0.69	0.65
L-Leucine	7.10	*7.95*	*7.88*	*7.19*	*0.85(12.0)*	*0.78(10.9)*	*0.09(1.3)*	471,825.89	472,451.13	150,803.45	1.24	1.02	1.77
L-Glutamic acid	**6.00**	**6.01**	**6.01**	**5.74**	**0,01(0.2)**	**0.01(0.2)**	**-0.26(-4.3)**	129,587.76	130,848.18	129,510.87	1.51	2.63	1.38
Phenylacetyl-L-glutamine	15.10	*16.41*	*16.08*	*16.19*	*1.31(8.7)*	*0.98(6.5)*	*1.09(7.2)*	109,072.62	109,755.39	109,691.28	2.52	2.20	2.51
Decanoyl-carnitine	19.30	*19.61*	*19.67*	*19.62*	*0.31(1.6)*	*0.37(1.9)*	*0.32(1.7)*	79,347.8	79,185.98	79,435.94	2.06	1.90	2.12

The metabolites with almost identical relative differences in isotopic ratios compared to the theoretical ratios for both correction methods are represented by numbers in italics, while those with greater differences are represented by bold numbers

^*^Experimental isotopic ratios were determined from peak picked data using the M/M + 1 ratio for ^13^C isotopologues. Numbers in bold are metabolites where more important differences are found in the estimations. **Difference = (Estimated data – Theoretical value). ***Percentage = (Estimated data – Theoretical value)/theoretical value.

Both LOESS and ComBat yielded modest improvements in isotopic ratio accuracy compared to uncorrected data. LOESS outperformed ComBat in 9 out of 13 compounds in terms of absolute deviation from the theoretical value.. However, ComBat introduced significant overcorrections in several cases (e.g., Pipecolic acid, Creatine), where the isotopic ratio deviated further from the theoretical reference than in the raw data. To further evaluate the correction performance, both absolute differences (in ratio units) and relative errors (percentage deviation from theoretical values) were compared. In most cases, the two criteria were concordant; meaning compounds with high absolute deviations also exhibited high relative errors. However, a few compounds with low theoretical ratios (e.g., Creatinine or L-Proline) showed small absolute deviations but relatively high relative errors, revealing that relying solely on absolute differences may underestimate the extent of correction bias in low-abundance contexts. Thus, both criteria are complementary and should be jointly considered to avoid misinterpretation. This analysis highlights that, isotopic ratios when theoretical expectations are known, can serve as internal quality control metrics for batch effect correction methods. They offer a chemical anchor point that allows researchers to assess not only the agreement of corrected values but also to detect overcorrection artifacts, as illustrated by ComBat in some cases. Using the paired Wilcoxon test, significant deviations from theoretical values were observed for both uncorrected and LOESS-corrected data (p = 0.013 for both), indicating a systematic bias. In contrast, no significant deviation was detected after ComBat correction (p = 0.068, α = 0.05), suggesting the absence of a systematic shift. The number of metabolites considered in this test being low (n = 13), the interpretations of the statistical test must be done with caution. However, the variability was quantified using absolute deviations from theoretical values. The median absolute errors were 0.31 (without correction), 0.37 (LOESS), and 1.09 (ComBat), while the mean absolute errors were 0.60, 0.58, and 0.99, respectively. These results indicate that ComBat correction was associated with substantially larger dispersion around the theoretical values compared to the other methods. These findings reflect two distinct aspects of performance. ComBat reduced systematic bias (no significant deviation, p = 0.068) but increased variability (median error = 1.09), whereas LOESS and uncorrected data exhibited smaller deviations (median errors = 0.37 and 0.31, respectively) but retained a significant systematic offset (p = 0.013). Overall, these results highlighted a trade-off between bias correction and precision: ComBat improved centering relative to theoretical values, while LOESS and uncorrected data maintained lower variability but remained systematically shifted. The convergence between absolute and relative criteria supports its robustness. The use of known isotopic ratios should be encouraged as a practical, compound-level tool for evaluating and validating batch correction methods in untargeted metabolomics workflows.

Taken together, the proposed workflow allowed highlighting key differences between LOESS and ComBat batch correction methods, offering valuable insights for selecting and evaluating these approaches. Multivariate analyses demonstrated that both methods effectively reduced batch effects, but the LOESS correction introduced more outliers, while achieving stronger batch-wise alignment. Numerical assessments, including inter-batch dispersion and coefficient of variation, confirmed that they both significantly improved data comparability, with LOESS providing better intra-batch consistency but slightly altering data structures. Univariate assessments on landmark compounds revealed that ComBat better preserved isotopic ratios integrity, whereas LOESS reduced variation more strongly. These findings underline the importance of selecting correction methods based on study objectives. LOESS could be recommended when the primary goal is minimizing batch effects at the risk of structural modifications, while ComBat is preferable for preserving intrinsic data properties. Beyond presenting this workflow, the purpose of this work was to illustrate it using a real LC/HRMS dataset, providing a step-by-step example case study. However, it may be of interest to test it with other datasets, especially those acquired using different analytical techniques, to evaluate its genericity in further work.

## Conclusion

Batch effects pose a significant challenge to data comparability and reliability in large-scale metabolomics studies. This work presents a workflow designed to assess the effectiveness and reliability of batch correction methods. By integrating a novel BCI parameter, multivariate analysis, and univariate evaluation using landmark compounds, the workflow provides a structured approach for determining the extent to which correction methods such as LOESS and ComBat help to improve data harmonization while preserving biochemical information. The proposed framework allows researchers to go beyond visual assessments, incorporating quantitative metrics to systematically compare correction methods, while ensuring that batch-wise variations are adequately mitigated without introducing new biases. By implementing this workflow, users can systematically evaluate batch correction reliability through a standardized protocol, thereby promoting reproducibility and enhancing the robustness of metabolomic analyses for better biological relevance.

## Supplementary Information

Below is the link to the electronic supplementary material.

Supplementary file1 (PDF 1179 KB)

## Data Availability

No datasets were generated or analysed during the current study.

## Code availability

All codes used in this work are publicly available in git repository at: https://github.com/Beeliada-Elfried/Integrated-workflow-for-univariate-and-multivariate-evaluation-of-batch-correction-reliability. Moreover, the dispersion indicators and visualization are available in the *dispersionIndicators* R package. Finally, all the methods will be soon available in Workflow4Metabolomics (Giacomoni et al., 2015) for a more extensive dissemination to end-users.
