# Supplementary material for: Integrated workflow for univariate and multivariate evaluation of batch correction reliability
Source: Metabolomics. 2026 Jul 23;22(4):131. doi: 10.1007/s11306-026-02453-1 (PMC13395890; doi:10.1007/s11306-026-02453-1)
Supplement: Supplementary file 1 — Supplementary file1 (PDF 1179 KB) [file 11306_2026_2453_MOESM1_ESM.pdf]

Supplemental Figure 1: Bivariate dispersion visualizations for different reference compounds, using QCs analyzed within nine batches on a large-scale study. The dot in the figure represents the different quality controls. Each convex hull represents an analytical batch and the covered surface represents the bivariate dispersion.

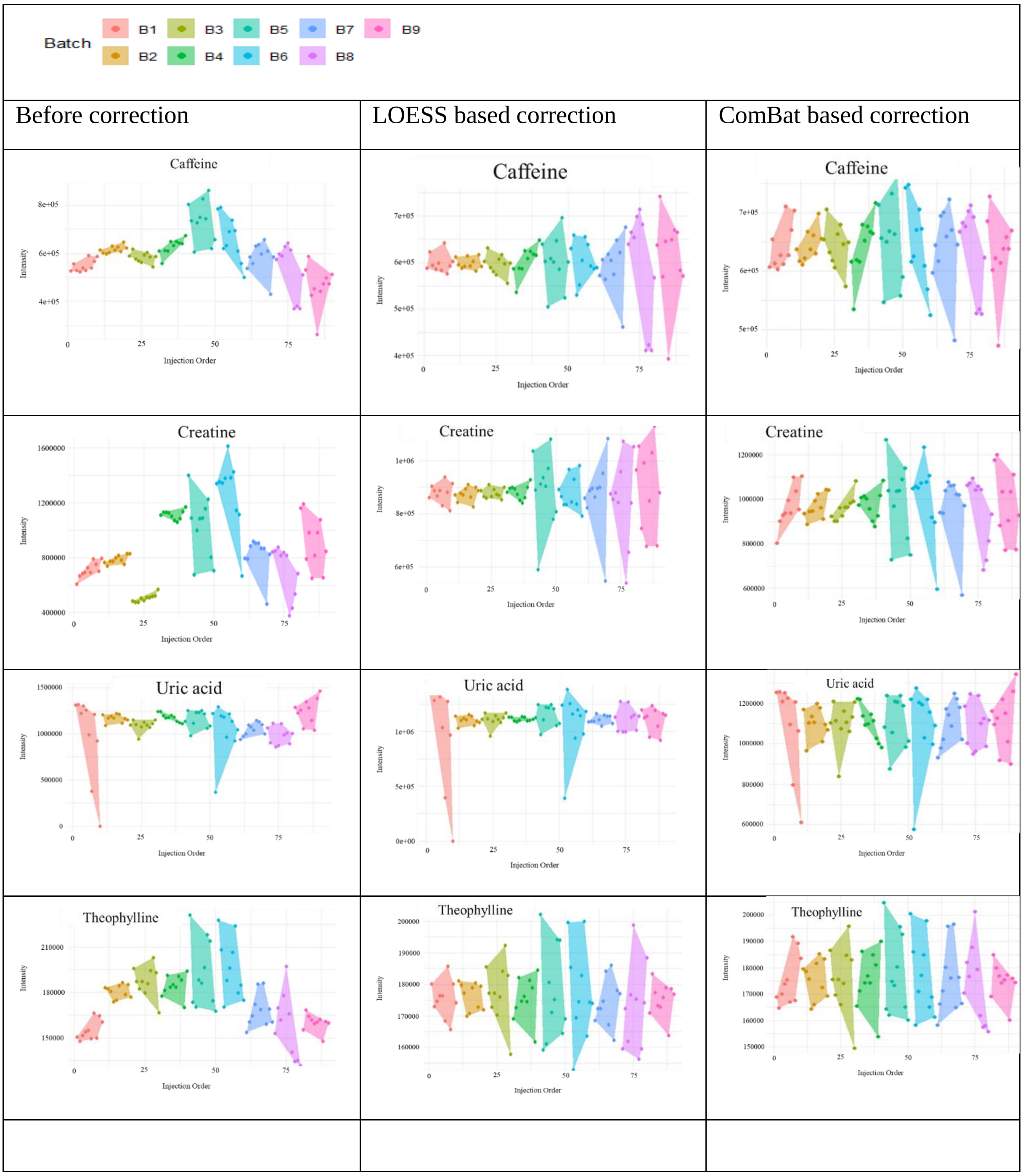

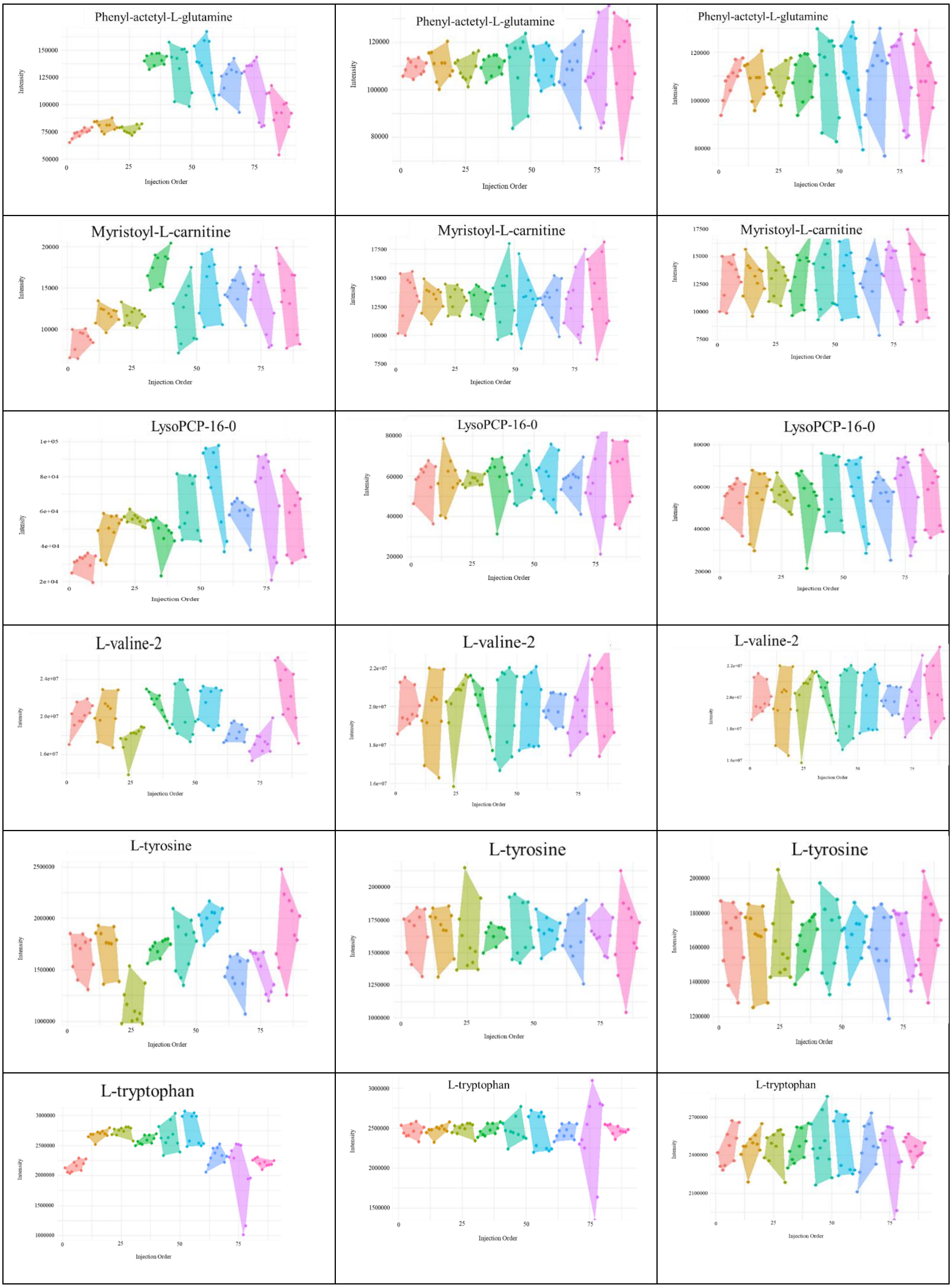

Phenyl-actetyl-L-glutamine
Intensity
Injection Order
Phenyl-actetyl-L-glutamine
Intensity
Injection Order
Phenyl-actetyl-L-glutamine
Intensity
Injection Order
Myristoyl-L-carnitine
Intensity
Injection Order
Myristoyl-L-carnitine
Intensity
Injection Order
Myristoyl-L-carnitine
Intensity
Injection Order
LysoPCP-16-0
Intensity
Injection Order
LysoPCP-16-0
Intensity
Injection Order
LysoPCP-16-0
Intensity
Injection Order
L-valine-2
Intensity
Injection Order
L-valine-2
Intensity
Injection Order
L-valine-2
Intensity
Injection Order
L-tyrosine
Intensity
Injection Order
L-tyrosine
Intensity
Injection Order
L-tyrosine
Intensity
Injection Order
L-tryptophan
Intensity
Injection Order
L-tryptophan
Intensity
Injection Order
L-tryptophan
Intensity
Injection Order

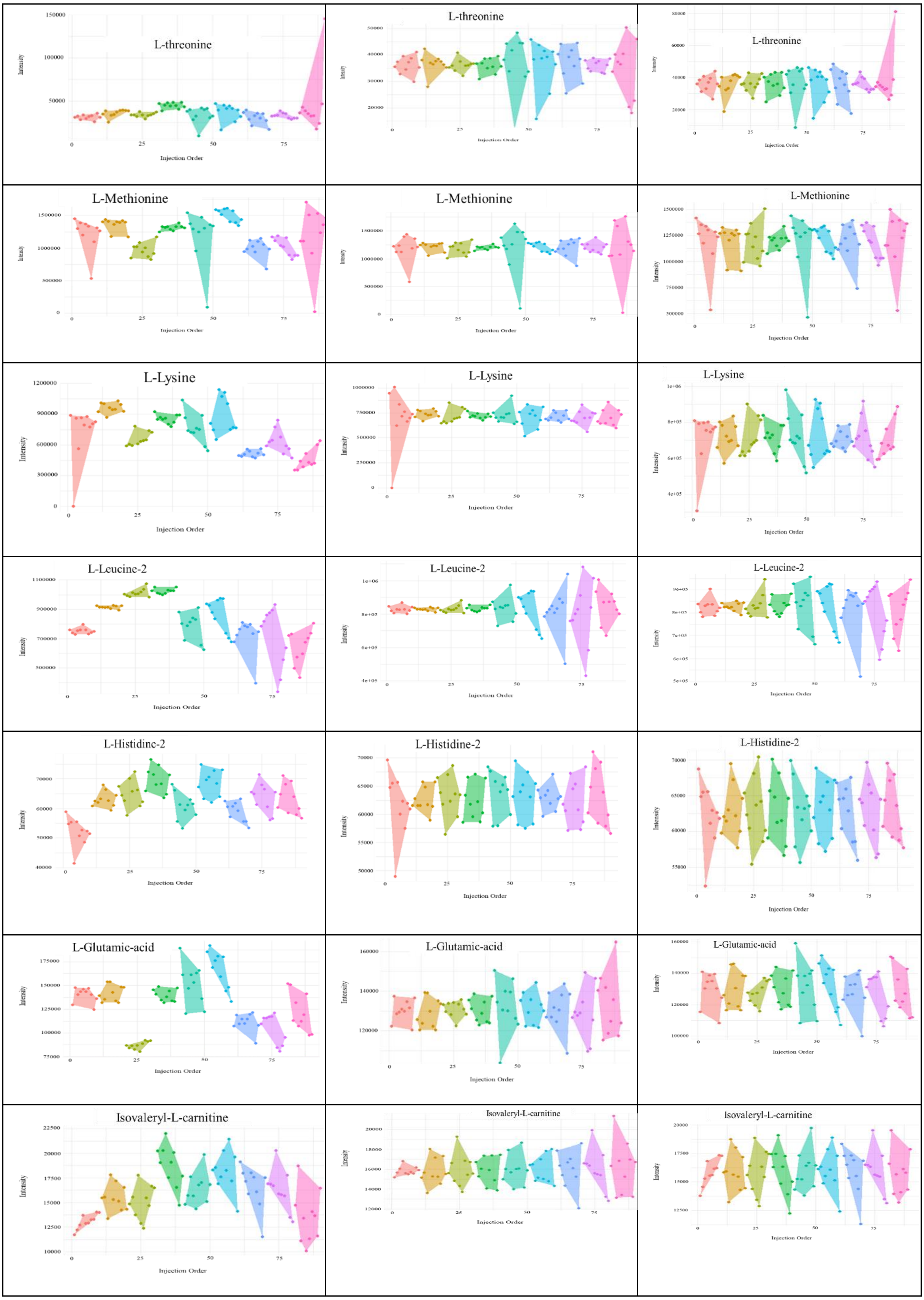
L-threonine
Intensity
Injection Order
L-threonine
Intensity
Injection Order
L-threonine
Intensity
Injection Order
L-Methionine
Intensity
Injection Order
L-Methionine
Intensity
Injection Order
L-Methionine
Intensity
Injection Order
L-Lysine
Intensity
Injection Order
L-Lysine
Intensity
Injection Order
L-Lysine
Intensity
Injection Order
L-Leucine-2
Intensity
Injection Order
L-Leucine-2
Intensity
Injection Order
L-Leucine-2
Intensity
Injection Order
L-Histidine-2
Intensity
Injection Order
L-Histidine-2
Intensity
Injection Order
L-Histidine-2
Intensity
Injection Order
L-Glutamic-acid
Intensity
Injection Order
L-Glutamic-acid
Intensity
Injection Order
L-Glutamic-acid
Intensity
Injection Order
Isovaleryl-L-carnitine
Intensity
Injection Order
Isovaleryl-L-carnitine
Intensity
Injection Order
Isovaleryl-L-carnitine
Intensity
Injection Order
